# Symptoms of Traumatic Encephalopathy Syndrome are Common in Community-Dwelling Adults

**DOI:** 10.1007/s40279-024-02029-w

**Published:** 2024-04-30

**Authors:** Douglas P. Terry, Anthony E. Bishay, Grant H. Rigney, Kristen Williams, Philip Davis, Jacob Jo, Scott L. Zuckerman

**Affiliations:** 1 Department of Neurological Surgery, Vanderbilt Sports Concussion Center, Vanderbilt University Medical Center, Suite 4340, 1500 21St Ave South, Nashville, TN 37206 USA; 2 School of Medicine, Vanderbilt University, Nashville, TN USA; 3 Department of Physical Medicine and Rehabilitation, Harvard Medical School, Boston, MA USA

## Abstract

**Background and Objectives:**

The consensus criteria for traumatic encephalopathy syndrome (TES), the possible in vivo clinical syndrome associated with significant repetitive head impacts, have only been minimally studied to date. This study examined the prevalence of the proposed core clinical features of TES in a sample of healthy adults.

**Methods:**

A cross-sectional survey study was conducted through ResearchMatch, a national health volunteer registry. Participants were assessed for symptoms of TES based on the 2021 consensus criteria, including prior repetitive head impacts and core clinical features. Additional health information (e.g., concussion history, psychological health, sleep, chronic pain) was also evaluated. The consensus proposed research criteria for TES (i.e., reporting at least one progressive core clinical feature of TES, as in progressive difficulties with episodic memory, executive functioning, or neurobehavioral dysregulation) were applied to the sample.

**Results:**

Out of 1100 participants (average age = 53.6 ± 17.7 years, 55% women), 34.6% endorsed one or more progressive core clinical features of TES. Participants with a significant history of contact sports (i.e., ≥ 5 years total, with ≥ 2 years in high school or beyond) had similar rates of endorsing a progressive core clinical feature of TES compared to those without significant histories of repetitive head impacts (36.4% vs 32.8%, respectively, *χ*^2^ = 0.52, *p* = 0.47). A significant history of repetitive head impacts in sports was not associated with endorsing a core clinical feature of TES in univariable or multivariable models (*p* > 0.47), whereas current depression/anxiety (odds ratio [OR] = 6.94), a history of psychiatric disorders (OR = 2.57), current sleep problems (OR = 1.56), and younger age (OR = 0.99) were significant predictors of TES status in a multivariable model. In a subsample of 541 participants who denied a lifetime history of contact sports, other forms of repetitive head impacts, and concussions, approximately 31.0% endorsed one or more progressive core clinical features of TES. Additionally, 73.5% of neurotrauma-naïve participants with current anxiety or depression reported at least one core progressive feature of TES, compared with 20.2% of those without clinically significant depression/anxiety symptoms.

**Conclusions:**

A considerable proportion of adults without a significant history of repetitive head impacts from sports endorsed core TES features, particularly those experiencing mental health symptoms. Having a significant history of contact sports was not associated with endorsing a core progressive clinical feature of TES, whereas other health factors were. These findings underscore the need for validating and refining TES criteria in samples with and without substantial neurotrauma histories.

**Supplementary Information:**

The online version contains supplementary material available at 10.1007/s40279-024-02029-w.

## Key Points

**Table:** 

This study is one of the first to examine the recently published consensus criteria for traumatic encephalopathy syndrome in adults. Over one third of the sample reported one or more progressive core clinical features of traumatic encephalopathy syndrome.
Having a significant history of contact sports was not associated with higher rate of endorsing a progressive core clinical feature of traumatic encephalopathy syndrome, whereas psychological difficulties and sleep problems were.
These data suggest the consensus criteria for TES may lack specificity, which has important clinical implications for providers who treat individuals with a substantial history of repetitive impacts such that it will be essential to discern whether the symptoms reported by patients are a consequence of their neurotrauma history or non-neurotrauma etiologies.

## Introduction

Chronic traumatic encephalopathy (CTE) and traumatic encephalopathy syndrome (TES) have recently moved to the forefront of the conversation about the safety of American football and boxing and their potential repercussions [1–3]. Definitions for TES (which we refer to as the possible in vivo clinical syndrome), as opposed to the postmortem neuropathological entity thought to be associated with repetitive head impacts, hereby referred to as CTE neuropathologic change have been proposed. However, the growing emphasis on research has revealed flaws in those definitions [4–8]. The non-specific nature of prior iterations of the TES criteria [8] has been demonstrated in research that shows a substantial minority [9] of the general population endorsed a constellation of clinical symptoms consistent with TES [10] (most notable those with psychiatric and other comorbidities) [11, 12]. Research has also suggested that a significant history of contact sport participation did not increase the likelihood of meeting the clinical criteria for TES compared to those without significant contact sport histories [10]. Furthermore, in a cohort of 85 retired professional contact sport athletes, meeting criteria for TES was not associated with years of professional play nor the number of games played professionally [13].

To improve diagnostic accuracy, a panel of expert clinician scientists met in 2019 and utilized a modified Delphi process to generate a new operational definition of TES for research purposes, which was published in 2021 [14]. These consensus TES criteria have only been minimally studied to date. For instance, a recent study investigated the consensus TES criteria in 176 professional fighters and found that 41% of the sample met TES criteria, and the likelihood of TES was associated with increasing age, a higher number of fights, younger age when starting to compete, and other factors [15]. However, studies examining the new criteria in various sports are more limited. [10, 13, 16]

The aim of this study is to examine the prevalence of TES symptoms in a group of community-dwelling healthy adults, as defined by the National Institute of Neurological Disorders and Stroke consensus criteria, and their association with a history of repetitive neurotrauma. We believe that it is imperative to examine the prevalence of the clinical symptoms of TES in a community-dwelling population to understand the potential specificity of these criteria. If the clinical symptoms of TES are common in samples *without* a substantial history of repetitive head impacts (due to non-head injury factors), then it will be important for healthcare providers to carefully and thoughtfully take these non-head injury factors into account when working with former athletes who present with TES-like symptoms to ensure that all differential diagnoses are considered in order to provide the appropriate medical care to former athletes.

## Methods

### Standard Protocol Approvals, Registrations, and Patient Consents

The study was approved by the Vanderbilt Institutional Review Board (VUMC IRB #230651). Written informed consent was obtained from all participants in the study.

### Participants and Procedures

A cross-sectional survey study was conducted. Recruitment for the study was done via ResearchMatch [17], a national health volunteer registry that was created by several academic institutions and supported by the US National Institutes of Health as part of the Clinical Translational Science Award program. ResearchMatch has a large population of volunteers who have consented to be contacted by researchers about health studies for which they may be eligible. Potential participants were invited to be part of a study titled “Assessing Brain Health in Adults.” There was no mention of sports, traumatic brain injuries, concussion, or CTE in the study information to reduce potential participation bias. All participants were at least 18 years of age. No other exclusion criteria were applied. There was no financial compensation associated with this study.

### Survey

The survey sent to participants assessed for all features of TES as defined in the consensus criteria [14]. Additional information participants were asked to provide general demographic information, concussion history, sports participation history, general health history, and any current symptoms of any condition as described in the following categories. All data were self-reported.

#### Repetitive Head Impacts

A detailed history of participants’ involvement in sport was queried. Participants reported each organized sport they competed in. For each sport they reported playing, they specified how many years they played at each level (i.e., number of years before high school, during high school, during college, semi-professionally, and professionally). Questions assessing other potential repetitive head impacts (e.g., military, law enforcement service, or domestic violence) were also included in the survey. For individuals who reported having experienced domestic violence or law enforcement/military service, further information was gathered about the number of times they engaged in combative training (in days), number of explosions/breaches they experienced overpressure, number of rounds of heavy weapons, number of controlled detonations, number of improvised explosive devices (IEDs), and number of instances in which they experienced domestic violence with strikes to the head.

#### Concussion History

The recently published TES consensus criteria do not include a history of concussion(s) as part of its research diagnostic criteria. However, we believed this was important to assess. The survey presented this definition of concussion: “*We define a concussion as a blow to the head or whiplash that caused any one or more of the following: (1) witnessed loss of consciousness (being “knocked out” and someone seeing it), (2) loss of memory for events immediately before and/or after the injury, or (3) feeling dazed and confused for at least 30s.”* Participants reported the number of concussions they experienced and the date of their most recent concussion.

#### Proposed Core Clinical Features of TES

The TES consensus criteria outline three core clinical features, which were assessed. Participants were asked if they had significant problems with (1) episodic memory (“significant problems with my memory for specific events that I have experienced, such as recent conversations or important things I have done in the past two weeks”), (2) executive functioning (issues with “planning things in my daily life; organizing my daily schedule; flexible thinking; inhibiting my impulses; shifting between tasks; multitasking; problem solving”), and (3) neurobehavioral dysregulation (“significant problem controlling my emotions and behavior” such as issues with explosiveness, impulsivity, rage, violent outbursts, having a “short fuse,” “mood swings”) for 1 year or more. Answer choices were: no; yes to some degree; and yes definitely. If participants endorsed either “yes” option, a follow-up question assessed if they feel this problem has become worse in the past year to assess the progressive nature of each feature.

#### Other Current Symptoms

Supportive features of TES were also assessed, such as dysarthria, ataxia, imbalance, and tremor over the past year (response options: never, rarely, sometimes, often, always). Lifetime history of anxiety (yes/no) and depression (yes/no) were also queried based on the participant endorsing if a healthcare provider told them they had either diagnosis. Current symptoms of depression and anxiety during the past 2 weeks were assessed using the Patient Health Questionnaire-9 [18] (PHQ-9) and the Generalized Anxiety Disorder-7 [19] (GAD-7). Functional status was gauged using the follow scale: independent, slightly reduced performance/functioning, definite impairment, not independent, and cannot participate (see Table 1 of the Electronic Supplementary Material for a detailed explanation of functional status). Though not a part of the TES consensus criteria, frequency of sleep difficulties (i.e., “trouble falling or staying asleep”), chronic pain (i.e., pain in one or more parts of my body”), and migraine headaches were assessed over the past year (response options: never, rarely, sometimes, often, always).

### Sport Exposure Criterion

Based on the TES consensus criteria, participants were placed into groups based on their history of involvement in high-exposure contact/collision sports that was likely associated with substantial repetitive head impacts. Consistent with Katz et al. [14], substantial exposure to contact sport (hereby referred to as the Sport Exposure Criterion) was defined in this study as having ≥ 2 years of contact sport participation during high school (or beyond) *and* ≥ 5 years of contact, collision, or combat sport participation during their lifetime. Football, soccer, lacrosse, boxing, hockey, rugby, martial arts, and wrestling were included and considered high-risk contact sports in this study. Participants who did not meet the Sport Exposure Criterion *and* denied other forms of repetitive head impacts were used as a comparison group. Participants who did not meet the Sport Exposure Criterion but endorsed a history of other repetitive head impacts (e.g., military service, law endorsement, domestic violence) were separated into a “Other Head Impacts” group and not included in the main statistical analyses given that the minimum threshold for these head impacts has not yet been established.

### Statistical Analyses

Descriptive statistics were conducted to provide an overview of the sample, including the proportions of individuals who met the Sport Exposure Criterion. Additionally, the proportions of the sample that reported each core clinical feature of TES were presented, along with the proportions of those who reported other health problems unrelated to TES. The χ^2^ analysis was employed to assess the relative proportions of individuals who fulfilled the core clinical features of TES, categorized based on whether they met the Sport Exposure Criterion versus those who did not meet this criterion or endorse other head impacts. In the same manner, a *χ*^2^ analysis was utilized to examine the proportions of individuals who manifested health issues unrelated to TES, grouped according to whether they met the Sport Exposure Criterion or not. Two binary logistic regressions were performed to predict the presence of one or more progressive core clinical features of TES using several independent variables. In both regressions, our primary independent variable was the Sport Exposure Criterion. The first regression included covariates that are independent of the consensus TES criteria (i.e., age, gender, chronic pain, and sleep problems). In the second model, we extended the variables from the first model and introduced psychiatric features, which are considered supportive features of the consensus TES criteria. It was important to ensure that these psychiatric features did not fully explain the TES core clinical features. We operationalized the psychiatric features by considering a history of psychiatric disorders (i.e., depression, anxiety, post-traumatic stress disorder, and substance abuse) as well as screening positively for current depression or anxiety (i.e., PHQ-9 ≥ 10 or GAD-7 ≥ 10). In a sub-analysis including individuals with no history of head impacts (i.e., no prior concussion history, as well as no contact sport exposure, military exposure, or history of head trauma from domestic violence), we present the proportions of those who reported TES features. These proportions are stratified based on the significant covariates included in our second multivariable model. All analyses were conducted using SPSS version 28.0 (IBM, Armonk, NY, USA).

### Data Availability

The survey, statistical analyses, and underlying data supporting the conclusions of this article will be made available by the authors to qualified researchers, without undue reservation.

## Results

### Sample Characteristics

**Fig. 1:**
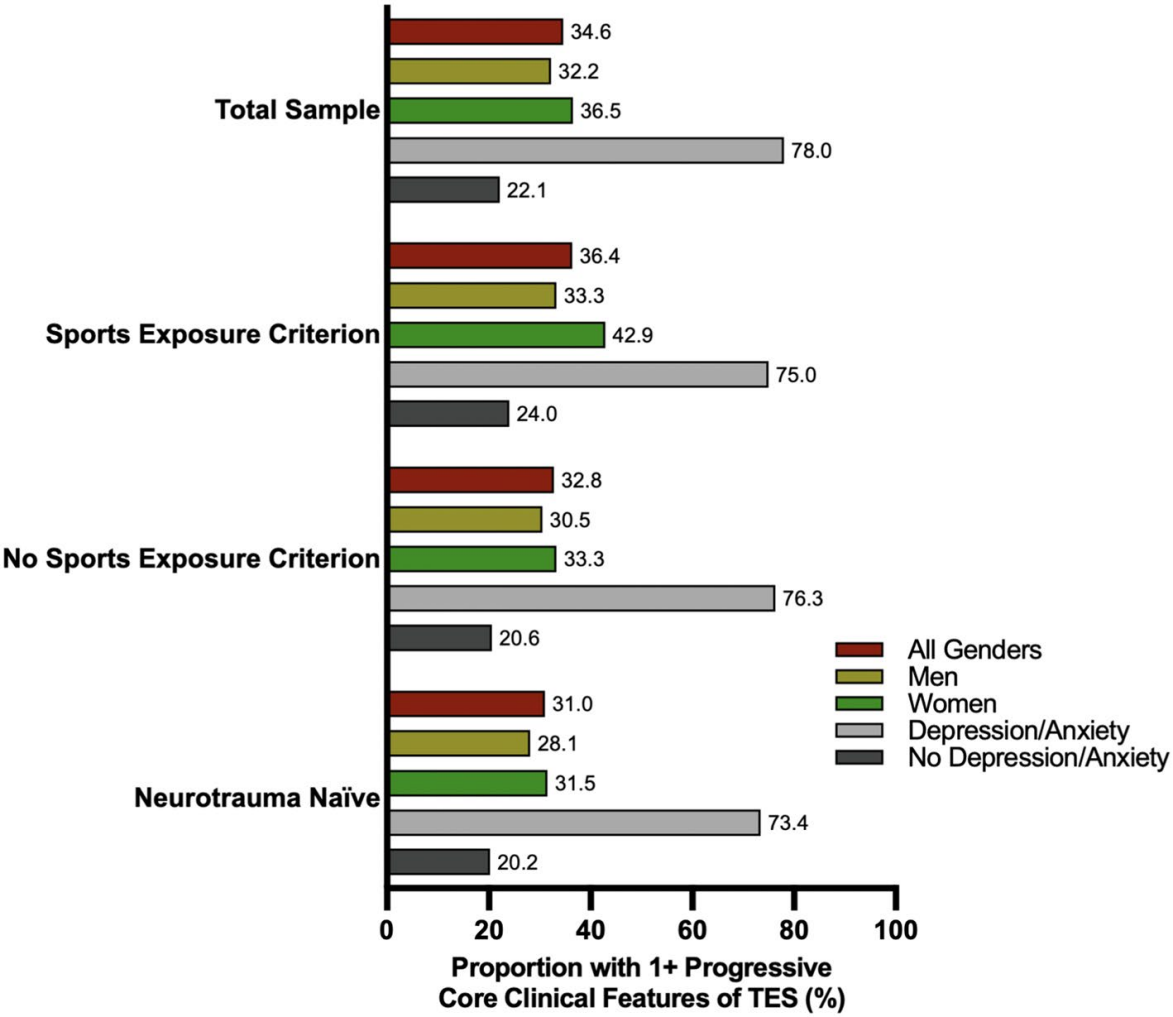
Proportion of sample who endorsed one or more core clinical features of traumatic encephalopathy syndrome (TES)

A total of 55,180 individuals were invited to participate in the study. Of these, 1743 unique participants consented to the study (3.16%), but several were excluded. Some (*n* = 425) completed less than 100% of the survey, including important variables of interest such as sport history, and were excluded from all analyses. Additionally, quality-control analyses revealed some participants (*n* = 52) who appeared to respond in an invalid manner (i.e., endorsed having *multiple* conditions, such as encephalocele, Rett’s disorder, multiple sclerosis, amyotrophic lateral sclerosis, and several others) and were subsequently excluded. Further, 12 individuals reported having a concussion over the past 6 months and 154 individuals reported having neurological conditions (*n* = 8 Parkinson’s disease, *n* = 3 Alzheimer’s disease, *n* = 37 epilepsy, *n* = 113 mild cognitive impairment, *n* = 25 dementia, *n* = 2 CTE). Given that symptoms of concussion and these neurological conditions can be associated with a variety of cognitive and psychological symptoms, we chose to exclude these individuals in an effort not to over-represent the proportion of people who experience TES-like symptoms (Fig. 1).

The final sample included 1100 participants (1.99% of those invited to participate). The mean age was 53.6 years (standard deviation = 17.7 years, range = 18.0–92.0 years). The sample was predominantly female (55.3%; male = 42.5%; transgender = 0.9%; other = 1.3%), white (89.5%; Black/African American = 4.7%, Asian = 1.9%, Native American = 0.6%; Other = 3.2%), and not Hispanic or Latino/a (94.9%; Hispanic or Latino/a = 5.1%). The average length of education in the sample was 16.7 ± 2.2 years (range: 8–19 years), with 41.9% of individuals holding an advanced degree (e.g., master’s or doctorate; *n* = 461). More than half were currently married (52.4%, never married = 21.1%, separated/divorced = 12.7%, living with partner = 9.2%, widowed = 4.6%). Regarding concussion history, 55.5% had no prior history of concussions, 19.7% reported one prior concussion, 10.8% reported two concussions, and 13.8% reported three or more concussions. Regarding repetitive head impacts, 9.0% (*n* = 99) met the Sport Exposure Criterion and 10.1% (*n* = 111) of participants who did not meet the Sport Exposure Criterion reported other forms of repetitive head impacts (Table 1).

**Table 1:** Sample demographics and head injury exposures

Demographics	*n* = 1100
Age	*M* = 53.6, Md = 56.0, SD = 17.7, IQR = 38.0–69.0, range = 18.0–92.0
Gender	
Men	*n* = 468, 42.5%
Women	*n* = 608, 55.3%
Transgender	*n* = 10, 0.9%
Other	*n* = 14, 1.3%
Race	
Black/African American	*n* = 52, 4.7%
White	*n* = 985, 89.5%
Asian	*n* = 21, 1.9%
Native American	*n* = 7, 0.6%
Other	*n* = 35, 3.2%
Ethnicity	
Hispanic or Latino/a	*n* = 56, 5.1%
Not Hispanic or Latino/a	*n* = 1044, 94.9%
Marital status	
Married	*n* = 576, 52.4%
Living with partner	*n* = 101, 9.2%
Separated/divorced	*n* = 140, 12.7%
Widowed	*n* = 51, 4.6%
Never married	*n* = 232, 21.1%
Education	
Eighth grade	*n* = 1, 0.1%
High school/GED	*n* = 52, 4.7%
Some college/technical degree/AA	*n* = 217, 19.7%
College degree	*n* = 369, 33.5%
Advanced degree (MS, PhD, MD)	*n* = 461, 41.9%
Concussion history	
0	*n* = 611, 55.5%
1	*n* = 216, 19.6%
2	*n* = 118, 10.7%
3+	*n* = 151, 13.7%
Missing	*n* = 4, 0.4%
Most recent concussion 6–12 months previously 1 or more years previously Missing	*N* = *485 with concussion history* *n* = 4, 0.8% *n* = 461, 95.1% *n* = 20, 4.1%
Repetitive neurotrauma	
Contact sport criteria (a total of 5 + years of contact sport exposure, with at least 2 years in high school or above)	*n* = 99, 9.0%
Other head impacts	(Participants may have endorsed > 1 of these)
Police with combative training	*n* = 7, 0.6%
Police with breaching	*n* = 3, 0.3%
Military/veterans with blast exposure	*n* = 36, 3.3%
Military/veterans with heavy weapon exposure	*n* = 36, 3.3%
Military/veterans with detonation exposure	*n* = 29, 2.6%
Military veterans with IEDs	*n* = 5, 0.5%
Domestic violence with head blows	*n* = 71, 6.5%
Total other head impacts (without meeting the Sport Exposure Criterion)	*n* = 111, 10.1%

*AA* Associate’s degree, *GED* General Educational Development diploma, *IEDs* improvised explosive devices, *IQR* interquartile range, *M* mean, *Md* median, *n* sample size, *SD* standard deviation

### Symptom Reporting Consistent with TES

Approximately one in three participants (34.6%) endorsed having one or more progressive core clinical features of TES (Table 2). The proportions of the sample that met core clinical features of TES criteria were as follows: memory problem = 24.4%, progressive memory problem = 22.9%, executive functioning problem = 28.4%, progressive executive functioning problem = 22.3%, neurobehavioral dysregulation = 31.8%, and progressive neurobehavioral dysregulation = 19.2%. Motor problems, which are discussed in the TES criteria as supportive features, were reported in 28.2% of the sample (balance = 17.5%, tremor = 13.2%, falls = 9.3%, and dysarthria = 5.8%). Regarding other health problems in the past year, 63.5% endorsed having chronic pain, 55.7% reported sleep problems, and 19.8% reported migraines. A total of 61.2% reported having a past history of psychiatric disorders (i.e., depression, anxiety, post-traumatic stress disorder, substance abuse), and 23.5% screened positively for depression or anxiety at the time of the survey (i.e., PHQ-9 ≥ 10 or GAD-7 ≥ 10). The majority of the sample reported being functionally independent (76.9%), while some reported subtle or mild functional limitations (19.1%) and the remainder reported more significant functional impairment (Table 3).

**Table 2:** Endorsement of each survey question used to assess TES features, consistent with the consensus criteria for TES (Katz et al. [13])

				Met sport exposure criterion				
Question	Total sample (*N* = 1100) (%)	Men (*N* = 468) (%)	Women (*N* = 608) (%)	No, and denied other head impacts (*N* = 890) (%)	Yes (*N* = 99) (%)	*χ*^2^	*P* value	Other head impacts (*n* = 111) (%)
Core clinical features of TES								
Memory								
“Significant problems with my memory for specific events that I have experienced, such as recent conversations or important things I have done in the past two weeks.”	23.8	21.6	25.5	22.0	29.3	2.68	0.10	38.7
Progressive memory problem	21.5	19.9	22.7	19.7	24.2	1.14	0.29	37.8
Executive functioning								
“Significant problems with executive functioning, which includes several cognitive skills and functions, including: planning things in my daily life, organizing my daily schedule, flexible thinking, inhibiting my impulses, shifting between tasks multitasking, problem solving.”	27.3	23.7	30.1	26.7	27.3	0.01	0.91	42.3
Progressive executive functioning problem	21.5	18.2	24.0	21.0	21.2	0.01	0.96	33.3
Neurobehavioral dysregulation								
“Significant problem controlling my emotions and behavior. Examples would be a significant problem with one or more of the following: explosiveness, impulsivity, rage, violent outbursts, having a “short fuse,” mood swings.”	31.0	31.2	30.9	29.4	38.4	3.37	0.07	45.0
Progressive neurobehavioral dysregulation	18.8	18.0	19.4	17.1	21.2	1.04	0.31	34.2
One or more progressive core clinical features	34.6	32.2	36.5	32.8	36.4	0.52	0.47	54.1

The above questions were presented to study participants verbatim and were based on common definitions of the terms used in the consensus definition

*TES* traumatic encephalopathy syndrome

**Table 3:** Endorsement of supportive TES symptoms and other health problems

Other health problems not related to TES	Total sample	Men	Women	Met Sport Exposure Criterion				
				No, and denied other head impacts	Yes	*χ*^2^	*P* value	Other head impacts
	*n* = 1100	*n* = 468	*n* = 608	*n* = 890	*n* = 99			*n* = 111
Sleep problems I have had trouble falling or staying asleep	55.7%	49.1%	59.5%	55.7%	53.5%	0.17	0.677	79.3%
Chronic pain I have had pain in one or more parts of my body	63.5%	59.8%	66.0%	62.2%	57.6%	0.82	0.364	57.7%
Migraines I have had migraine headaches	19.8%	9.8%	27.5%	19.1%	17.2%	0.22	0.642	27.9%
Supportive TES symptoms	*n* = *1100*	*n* = *468*	*n* = *608*	*n* = *890*	*n* = *99*			*n* = *111*
Dysarthria I have had difficulty with my speech, such as slurred or slow speech	5.8%	6.4%	5.3%	5.2%	8.1%	1.46	0.226	9.0%
Balance I have noticed difficulties or problems with my balance and my ability to walk	17.5%	16.0%	18.8%	15.7%	20.2%	1.31	0.252	29.7%
Falls I have lost my balance and fallen	9.3%	7.1%	10.5%	8.4%	8.1%	0.01	0.906	17.1%
Tremor I have a tremor (such as hand shaking)	13.2%	15.0%	11.5%	13.0%	11.1%	0.29	0.588	16.2%
Any motor problem	28.2%	27.8%	28.1%	26.3%	29.3%	0.41	0.521	42.3%
	*n* = *1100*	*n* = *468*	*n* = *608*	*n* = *890*	*n* = *99*			*n* = *111*
History of depression	47.5%	39.7%	51.8%	47.6%	37.4%	3.77	0.052	55.0%
History of anxiety	46.7%	36.5%	52.8%	48.2%	28.3%	14.22	** < 0.001**	51.4%
History of psychiatric disorders (i.e., depression, anxiety, PTSD, substance abuse)	61.2%	51.9%	66.8%	60.7%	52.5%	2.46	0.117	73.0%
	*n* = *1100*	*n* = *404*	*n* = *549*	*n* = *890*	*n* = *99*			*n* = *111*
PHQ-9 ≥ 10	21.3%	18.4%	22.4%	19.2%	23.2%	0.91	0.339	36.0%
GAD-7 ≥ 10	11.9%	10.3%	12.2%	10.7%	12.1%	0.19	0.660	21.6%
Current depression or anxiety (i.e., PHQ-9 ≥ 10 or GAD-7 ≥ 10)	23.5%	19.9%	25.2%	21.8%	24.2%	0.31	0.578	36.9%
Functional status	*n* = *1100*	*n* = *468*	*n* = *608*	*n* = *890*	*n* = *99*			*n* = *111*
Independent	76.9%	77.6%	74.5%	78.8%	65.7%	24.57	** < 0.001**	55.9%
Slightly reduced performance/functioning	19.1%	15.6%	19.7%	16.7%	18.2%			29.7%
Definite impairment	5.5%	5.6%	4.4%	3.5%	12.1%			13.5%
Not independent	1.1%	0.9%	1.3%	0.9%	3.0%			0.9%
Cannot participate	0.2%	0.4%	0.0%	0.1%	1.0%			0.0%

Sleep problems, chronic pain, and motor symptoms were considered to be present if the participant rated them “sometimes,” “often,” or “always” over the past year

Regarding functional status, participants were given brief definitions designed to correspond to the five levels of functional status discussed in the consensus criteria^21^ [i.e., (i) independent; (ii) subtle/mild functional limitations; (iii) mild dementia (i.e., impairment in instrumental activities of daily living); (iv) moderate dementia (i.e., not independent and needs assistance with basic activities of daily living); and (v) severe dementia (i.e., cannot participate in functions outside the home)] and chose the one they felt was most appropriate

Bold denotes *p* < 0.05

*GAD-7* General Anxiety Disorder-7, *PHQ-9* Patient Health Questionnaire-9, *PTSD* post-traumatic stress disorder, *TES* traumatic encephalopathy syndrome

The Sport Exposure Criterion group was compared to the group that did not meet the Sport Exposure Criterion (and denied other repetitive head impacts) on a variety of outcomes. There were no statistically significant differences between the proportion of the groups who endorsed the core clinical features of TES (all *p *values > 0.05; Table 2). Between those who met the Sport Exposure Criterion (*n* = 99) and those who did not (*n* = 890), those who met the Sport Exposure Criterion were *less* likely to endorse a history of an anxiety disorder (28.3% vs 48.2%; *χ*^2^ = 14.22, *p* < 0.001) compared with those who did not. There were no group differences on other outcome metrics such as current depression/anxiety symptoms, motor symptoms, chronic pain, sleep problems, or migraines (Table 3). In terms of functional status, those who met the Sport Exposure Criterion showed a difference in functional status compared with those who did not meet the criterion (*χ*^2^ = 24.57, *p* < 0.001), such that it those who met the Sport Exposure Criterion had worse functional status (Table 3).

### Predictors of TES Symptoms in the General Population

In a series of separate univariable logistic regressions predicting TES symptoms, significant predictors were: current psychiatric symptoms [odds ratio (OR) = 12.53], history of psychiatric disorders (OR = 5.47), migraine headaches (OR = 2.81), sleep problems (OR = 2.63), any motor problems (OR = 2.44), chronic pain (OR = 2.26), number of prior concussions (OR = 1.18), and younger age (OR = 0.97; Table 4). Gender and the Sport Exposure Criterion were not significant predictors in the unadjusted univariable analyses (Table 4).

**Table 4:** Univariable logistic regressions predicting the presence of one or more progressive core clinical features of traumatic encephalopathy syndrome

	Univariable						
	*B*	SE	Wald	*df*	*P* value	OR	95% CI for OR
Age	− 0.03	0.00	79.70	1	** < 0.001**	0.97	0.96–0.97
Gender (reference = women)	0.19	0.13	2.18	1	0.140	1.21	0.94–1.56
Sport Exposure Criterion	0.16	0.22	0.52	1	0.472	1.17	0.76–1.81
Chronic pain (sometimes or greater)	0.82	0.14	34.07	1	** < 0.001**	2.26	1.72–2.98
Sleep problems (sometimes or greater)	0.97	0.13	51.65	1	** < 0.001**	2.63	2.02–3.42
Number of prior concussions	0.16	0.04	17.63	1	** < 0.001**	1.18	1.09–1.27
Migraine headaches (sometimes or greater)	1.03	0.16	44.68	1	** < 0.001**	2.81	2.08–3.81
Any motor problem (sometimes or greater)	0.89	0.14	41.91	1	** < 0.001**	2.44	1.86–3.20
History of psychiatric disorders	1.70	0.16	116.22	1	** < 0.001**	5.47	4.02–7.45
Current depression or anxiety	2.53	0.17	217.16	1	** < 0.001**	12.53	8.95–17.54

All clinical variables are coded as binary: no/absent or yes/present. Current depression or anxiety refers to a score of 10 or more on the Patient Health Questionnaire-9 (PHQ-9) or Generalized Anxiety Disorder-7 (GAD-7)

Bold denotes *p* < 0.05

*CI* confidence interval, *OR* odds ratio, *SE* standard error

The first multivariable logistic regression model was significant in predicting TES symptoms, *χ*^2^ (5) = 116.05, *p* < 0.001. The model’s variables explained 15.8% of the variance in TES symptoms (Nagelkerke *R*^2^ = 0.158). The significant predictors were sleep problems (OR = 2.18), chronic pain (OR = 1.98), and younger age (OR = 0.97); gender and the Sport Exposure Criterion were not significant predictors of TES symptoms. The second model, which added psychological factors, was also significant (*χ*^2^ (7) = 274.99, *p* < 0.001), and explained 34.6% of the variance of TES symptoms (Nagelkerke *R*^2^ = 0.346). Significant predictors for the second model (listed in order of magnitude from largest to smallest) included: current depression or anxiety (OR = 6.94), a history of psychiatric disorders (OR = 2.57), sleep problems (OR = 1.56), and younger age (OR = 0.99). Gender, chronic pain, and the Sport Exposure Criterion were again not significantly associated with TES symptoms (Table 5).

**Table 5:** Multivariable logistic regression predicting meeting criteria for traumatic encephalopathy syndrome

	Multivariable model 1							Multivariable model 2						
	B	SE	Wald	df	*P*-value	OR	95% CI for OR	B	SE	Wald	df	*P*-value	OR	95% CI for OR
Age	− 0.03	0.00	51.35	1	** < 0.001**	0.97	0.96–0.98	− 0.01	0.05	6.43	1	**0.011**	0.99	0.98–1.00
Gender (reference = women)	0.15	0.16	0.93	1	0.336	0.86	0.63–1.17	0.14	0.17	0.62	1	0.431	0.87	0.62–1.23
Sport Exposure Criterion	0.11	0.25	0.18	1	0.669	1.11	0.69–1.80	0.20	0.28	0.51	1	0.476	1.22	0.71–2.09
Chronic pain (sometimes or greater)	0.68	0.16	17.98	1	** < 0.001**	1.98	1.44–2.71	0.29	0.18	2.57	1	0.109	1.33	0.94–1.89
Sleep problems (sometimes or greater)	0.78	0.16	25.39	1	** < 0.001**	2.18	1.61–2.95	0.44	0.17	6.65	1	**0.010**	1.56	1.11–2.18
History of psychiatric disorders	–	–	–	–	**–**	–	–	0.94	0.19	25.60	1	** < 0.001**	2.57	1.78–3.70
Current depression or anxiety	–	–	–	–	**–**	–	–	1.94	0.20	91.03	1	** < 0.001**	6.94	4.66–10.33

Bold denotes *p* < 0.05

*CI* confidence interval, *OR* odds ratio, *SE* standard error

### Sub-Analysis of Those with No History of Concussions or Repetitive Head Impacts

A post-hoc analysis showed that 541 participants denied a lifetime history of head impacts (i.e., those with no prior concussion history, no contact sports exposure/military exposure, and no history of head trauma from domestic violence). Approximately 31.0% of this neurotrauma-naïve sample endorsed one or more core features of TES (men = 28.1%, women = 31.5%; Table 6). There were no gender differences in the endorsement of the three TES core features. Participants who reported having motor problems were more likely to report all core clinical features of TES, except progressive neurobehavioral dysregulation, compared with those without motor problems (Table 6). Participants who endorsed a history of a psychiatric disorder were more likely to report each of the core clinical features of TES compared with those who did not endorse a history of psychiatric disorder (all *p* < 0.001). For instance, over 40% of neurotrauma-naïve participants with a lifetime psychiatric history reported at least one core progressive feature of TES (42.2%), compared with 14.6% of those with no history of psychiatric disorder (Table 6). Finally, those with current depression or anxiety were more likely to endorse each of the core clinical features of TES compared with those who did not endorse current depression/anxiety (i.e., memory, executive functioning, and neurobehavioral dysregulation; all *p* < 0.001). About three in four neurotrauma-naïve participants with current anxiety or depression reported at least one core progressive feature of TES, compared with one in five of those without clinically significant depression/anxiety symptoms (73.4% vs 20.2%; Table 6**)**.

**Table 6:** Sub-analysis of those with no history of head impacts (i.e., those with no prior concussion history, sports exposure, military exposure, or history of head trauma from domestic violence)

	Gender (*n* = 530)				Any motor problem (*n* = 541)				History of psychiatric disorders (*n* = 541)				Current depression or anxiety (*n* = 541)			
Core clinical features of TES	Men (*n* = 194) (%)	Women (*n* = 336) (%)	*χ*^2^	*P* value	No (*n* = 418) (%)	Yes (*n* = 123) (%)	*χ*^2^	*P* value	No (*n* = 221) (%)	Yes (*n* = 320) (%)	*χ*^2^	*P* value	No (*n* = 432) (%)	Yes (*n* = 109) (%)	*χ*^2^	*P* value
Memory	16.0	19.9	1.28	0.258	15.1	33.3	20.41	** < 0.001**	11.8	24.4	13.39	** < 0.001**	13.0	44.0	54.12	** < 0.001**
Progressive memory problem	14.5	17.3	0.71	0.400	13.0	30.9	21.52	** < 0.001**	9.1	22.6	16.71	** < 0.001**	10.7	42.6	62.16	** < 0.001**
Executive functioning	19.6	24.4	1.63	0.202	21.1	32.5	6.92	**0.009**	8.1	34.4	49.80	** < 0.001**	15.0	57.8	88.08	** < 0.001**
Progressive executive functioning problem	15.5	20.2	1.86	0.173	16.7	27.6	7.27	**0.007**	5.9	28.4	42.83	** < 0.001**	10.2	55.0	112.80	** < 0.001**
Neurobehavioral dysregulation	24.7	25.6	0.05	0.828	23.4	36.6	8.44	**0.004**	8.6	38.8	61.12	** < 0.001**	17.6	61.5	86.17	** < 0.001**
Progressive neurobehavioral dysregulation	16.1	16.7	0.03	0.857	15.6	22.8	3.43	0.064	4.5	25.9	41.85	** < 0.001**	8.4	52.3	117.83	** < 0.001**
One or more progressive core features	28.1	31.5	0.68	0.410	26.7	45.5	15.77	** < 0.001**	14.6	42.2	46.24	** < 0.001**	20.2	73.4	114.93	** < 0.001**

Bold denotes *p* < 0.05

*TES* traumatic encephalopathy syndrome

## Discussion

This study examined the new consensus criteria for TES in a sample of community-dwelling adults from the general population. Establishing the specificity of these criteria (i.e., the likelihood of individuals who truly do not have TES as screening negative for TES based on the operational definition) is essential given that there may be other factors that contribute to an individual having a core clinical feature. In this study, approximately one third of respondents exhibited one or more progressive core clinical features of TES. Surprisingly, meeting the criteria for having a substantial history of involvement in high-exposure contact/collision sports impacts was not associated with a higher likelihood of having a core clinical feature of TES. Interestingly, individuals with a significant history of contact sports were *less* likely to have a history of anxiety, though the contact sports group was more likely to have worse current functional status. In a multivariable logistic regression model, a history of substantial contact sport exposure was not predictive of endorsing one or more core clinical features of TES. Instead, significant predictors for endorsing a core clinical feature of TES included screening positively for current depression or anxiety, having a history of psychiatric disorders, and reporting current sleep problems. Importantly, 19.1% of individuals reported symptoms consistent with TES in the absence of a history of concussions, contact sports, and other forms of repetitive head impacts (28.1% of men and 31.5% of women). In this sub-analysis of participants with no history of head impacts, 73.4% of individuals with current symptoms of anxiety or depression endorsed having one or more core features of TES.

Currently, validated criteria for the *clinical* diagnosis of TES in living individuals do not exist, although multiple research groups have proposed operational definitions that are meant to be used for research purposes [7, 14, 15]. Relatively few studies have been conducted examining base rates of TES-like symptomatology in the general population, and even fewer have examined the newer consensus criteria for TES. In one recent study examining a large sample of the general population collected in the early 2000s [9], 6.6–11.9% of individuals met the symptom-based TES criteria based on a prior TES definition [20]. Similar to the results in our current study, this prior study [9] found that up to 89% of individuals who reported having chronic pain, a mood disorder, or a recent history of suicidality endorsed at least one core clinical feature of TES, suggesting that the likelihood of having TES-like symptomatology can be high in individuals with physical and psychological difficulties. Similarly, men with intermittent explosive disorder from the general population also had a high likelihood of reported symptoms similar to TES, with up to 65% meeting criteria for TES based on a prior operational definition [11], further highlighting the possibility for high rates of misdiagnosis because of overlapping symptom profiles with common treatable conditions.

Prior research examining substantial sport exposure and its association with meeting the clinical features of TES has also yielded similar findings to this study, suggesting that a significant history of contact sport exposure was not associated with a higher likelihood of having a core clinical feature of TES. For example, in a study investigating the TES features in a sample of men, exposure to 6 + years of contact sports was not associated with a higher likelihood of having the TES features compared to those with 0–5 years of contact sport exposure [10]. However, predictors of the core clinical features of TES in this prior study notably included sleep difficulties (OR = 6.68) and chronic pain (OR = 3.29)—regardless of neurotrauma history [10]—similar to the current study. These findings are also corroborated in studies investigating professional athletes, as the number of prior concussions, years of professional play, and the number of games played professionally were not associated with meeting criteria for TES [13]. Taken together, these studies highlight that TES-like symptoms are common in the general population, especially those with idiopathic health problems such as chronic pain, sleep issues, and mental health problems. The common nature of these symptoms in the general population is essential to take into account when working with former athletes, who are assumed to experience these same idiopathic health problems. Given the association between these health problems and TES-like symptoms, there is a high potential for former contact sport athletes and their healthcare providers to accidentally misattribute the etiology of the core TES features to prior head impacts instead of these co-occurring health issues, leading to a potential misdiagnosis of TES. While the consensus criteria for TES specifies that the core clinical features must not be fully accounted for by other disorders [14], it is unclear how this may be accomplished.

One interesting observation was that participants who had a significant history of contact sports appeared to report lower rates of functional independence than those without significant contact sport histories/other repetitive head impacts. In this study, the etiology of these functional difficulties is unclear given that this was a self-reported rating of an individual’s ability to complete activities of daily living for any reason (see ESM). Participants may have endorsed functional limitations because of any health issue, including but not limited to physical functioning, cognitive issues, and mental health issues. Given that the contact sport and control group did not differ on any other variables examined in this study, it is unclear what may have driven this effect.

### Implications and Future Research

The present study was not designed to make assertions regarding the rates of a diagnosis of TES within the general population, but rather, to establish the prevalence of self-report problems consistent with the proposed core clinical features of TES in the general population (including those who are neurotrauma naïve) and examine whether a significant history of contact sport exposure was associated with higher rates of these problems. This study serves as a foundation for establishing the base rate of symptoms consistent with the core clinical features of TES, which will be particularly necessary for medical providers. Given that approximately one third of the sample in this dataset reporting experiencing one or more core clinical feature of TES, healthcare practitioners working with individuals who have a history of repetitive head impacts will be tasked with discerning whether the symptoms reported by patients are a consequence of their neurotrauma history or non-neurotrauma etiologies. The potential repercussions of misattribution of symptoms in this context are substantial and have the capacity to result in significant patient harm. Additionally, future studies may be needed to refine the diagnostic criteria for TES further, given that a significant history of contact sports was not associated with a higher likelihood of endorsing a core clinical feature of TES in addition to the non-specific nature of these problems that were somewhat commonly endorsed in the general population (include those without any prior neurotrauma histories). It is also essential to establish if the clinical criteria of TES are related to CTE neuropathologic change, which is beyond the scope of this study. However, in a study examining 152 deceased contact sport athletes aged younger than 30 years who were symptomatic before death, nearly 60% of the sample did not have neuropathological evidence of CTE [21]. Further, retrospective clinical evaluations with next of kin showed that there were no differences in the severity of cognitive difficulties, apathy, depression, impulsiveness, and aggression between those who were positive for CTE neuropathologic change compared to those who did not have evidence of CTE neuropathologic change (Table 2 of the ESM) [21], leading the authors of this study to note that “symptoms are not specific to low-stage CTE” (p. 1047).

### Limitations

The current study has several limitations that must be acknowledged. First, data were collected via a structured survey. All data are self-reported. While effective for large-scale data collection, they may introduce response bias and lack the depth compared with gold standard clinical assessments or medical records validation. Second, self-selection bias among participants who voluntarily engaged in our study challenges the broad representativeness of our results. The majority of this sample was White and non-Hispanic, thereby reducing the generalizability of the results to other demographic groups. Additionally, 75% of respondents are highly educated with at least a college degree, which may limit the generalizability of this study. However, as prior studies have indicated that lower level education is associated with worse cognitive functioning [22] as well as mental health problems including anger/aggression, [23] the core clinical features of TES may be more prevalent in less educated samples. Similarly, given that individuals who have experienced systematic racism may more commonly experience mental health difficulties, [24] a more diverse sample may more commonly experience the core clinical features of TES. Third, this study did not employ formal neuropsychological assessments to determine a decline in objective cognitive functioning. Future efforts should incorporate clinical examinations by trained professionals, corroborate symptoms with family members, and include formal cognitive testing. Fourth, the cross-sectional design limits our ability to understand temporal dynamics and causal relationships. Fifth, our findings’ accuracy is dependent on participants’ recollection, influenced by potential recall bias. Last, this study employed a new consensus-based definition of TES that is comparatively understudied and operationalizes TES differently than previous definitions. Several studies discussed above such as Iverson and Gardner [11] used the Montenigro et al. operational definition of TES, [20] which limits the comparability between studies.

## Conclusions

In a sample of 1100 community-dwelling older adults, 34.6% endorsed having one or more of the proposed progressive core clinical features of TES. Further, about one in five individuals without any prior repetitive head impacts or concussions endorsed having a core feature of TES, and 73.4% of this neurotrauma-naïve subsample who also had significant mental health symptoms endorsed having a core feature of TES. Individuals with a substantial history of repetitive head impacts from contact sports were *not* more likely to have a proposed core clinical feature of TES compared to those without significant contact sport histories in a variety of analyses, while those with mental health problems and general health issues (e.g., sleep difficulties) had higher rates of endorsing at least one core clinical feature of TES. Taken together, these findings suggest that core clinical features of TES are common in the general population, especially among individuals with mental health problems. Future work should aim to validate and potentially refine the TES consensus criteria and delineate predictors of TES in people exposed to repetitive head impacts from the symptoms that are commonly experienced by individuals without significant neurotrauma histories.

## Supplementary Information

Below is the link to the electronic supplementary material.

Supplementary file1 (DOCX 17 KB)

## Declarations

### Availability of data and material

Data are available to qualified investigators on reasonable request.
