# Supplementary material for: Symptoms of Traumatic Encephalopathy Syndrome are Common in Community-Dwelling Adults
Source: Sports Med. 2024 Apr 30;54(9):2453–65. doi: 10.1007/s40279-024-02029-w (PMC11393129; doi:10.1007/s40279-024-02029-w)
Supplement: Supplementary file 1 — Supplementary file1 (DOCX 17 KB) [file 40279_2024_2029_MOESM1_ESM.docx]

**Symptoms of Traumatic Encephalopathy Syndrome are Common in Community-Dwelling Adults**

Douglas P. Terry, PhD^1*^

Grant H. Rigney, MSc^2^

Anthony E. Bishay, BS^3^

Kristen Williams, MS, LAT, ATC ^1^

Philip Davis ^1^

Jacob Jo, BA^1,3^

Scott L. Zuckerman, MD, MPH ^1^

**Supplement Table 1**

*Functional Status Question*

With regard to your:

- Job
- Household responsibilities
- Family, social, and community roles

Which of the follow best describes your current level of functioning? (Select one. For definitions, see below)

| 🞏 | I am independent.   - I am able to engage in hobbies and intellectual activities at my usual levels. - I am fully independent in my activities. |
| --- | --- |
| 🞏 | I have slightly reduced performance.   - I have slight problems in hobbies and intellectual interests. - I am mostly independent but may be more challenged in some *advanced activities.*** - I am fully independent in *basic activities.** |
| 🞏 | I have definite impairment of *advanced activities.***   - I am engaged in some home, family, social, and community activities. - I abandon more difficult activities. - I need cues for some *basic activities.** |
| 🞏 | I am not independent but can be taken to some functions outside the home.   - I can do only simple chores. - I have very restricted interests. - I need assistance with *basic activities.** |
| 🞏 | I cannot participate in functions outside the home.   - I have impaired *basic activities.** - I am not independent with self-care. - I am frequently incontinent. |

| ***Basic activities are related to:** | ****Advanced activities are related to:** |
| --- | --- |
| Personal hygiene and grooming (e.g., brushing, combing, styling hair) | Managing money (e.g., paying bills, completing taxes) |
| Toilet hygiene (e.g., getting to the toilet, cleaning oneself, and getting back up) | Cleaning and maintaining the house |
| Bathing/showering | Preparing meals |
| Dressing | Shopping for groceries and necessities |
| Self-feeding | Medication management |
| Functional mobility (e.g., ability to walk, get in and out of bed, get into and out of a chair) | Transportation within the community (e.g., driving, using public transportation) |
|  | Using the telephone (mobile or landline) |
